# Timing as an intrinsic property of neural networks: evidence from *in vivo* and *in vitro* experiments

**DOI:** 10.1098/rstb.2012.0460

**Published:** 2014-03-05

**Authors:** Anubhuti Goel, Dean V. Buonomano

**Affiliations:** Departments of Neurobiology and Psychology, and Integrative Center for Learning and Memory, University of California, 695 Young Drive, Gonda, Los Angeles, CA 90095, USA

**Keywords:** neural dynamics, temporally selective neurons, state-dependent network model, short-term plasticity

## Abstract

The discrimination and production of temporal patterns on the scale of hundreds of milliseconds are critical to sensory and motor processing. Indeed, most complex behaviours, such as speech comprehension and production, would be impossible in the absence of sophisticated timing mechanisms. Despite the importance of timing to human learning and cognition, little is known about the underlying mechanisms, in particular whether timing relies on specialized dedicated circuits and mechanisms or on general and intrinsic properties of neurons and neural circuits. Here, we review experimental data describing timing and interval-selective neurons *in vivo* and *in vitro*. We also review theoretical models of timing, focusing primarily on the state-dependent network model, which proposes that timing in the subsecond range relies on the inherent time-dependent properties of neurons and the active neural dynamics within recurrent circuits. Within this framework, time is naturally encoded in populations of neurons whose pattern of activity is dynamically changing in time. Together, we argue that current experimental and theoretical studies provide sufficient evidence to conclude that at least some forms of temporal processing reflect intrinsic computations based on local neural network dynamics.

## 1. Introduction

The dynamic nature of our natural environment and the need to move and anticipate events in this environment provided strong evolutionary pressures for the nervous system to process temporal information and generate timed responses. Additionally, later in evolution the temporal features of animal vocalizations came to play a fundamental role in social communication [1–3]. As a result of these evolutionary pressures, the human brain is exquisitely capable of processing temporal information and generating temporal patterns. Indeed, the sophistication of temporal processing is well illustrated by the observation that humans can reduce communication to a purely temporal code, as occurs when people communicate using Morse code. Despite the obvious importance of temporal processing to communication, learning, cognition, and sensory and motor processing, even the most basic mechanisms of how animals discriminate simple intervals or generate timed responses remains unknown.

A key question in the debate of how the brain tells time relates to whether timing should be viewed as ‘dedicated’—relying on specialized and centralized mechanisms—or ‘intrinsic’—relying on local and general properties of neural circuits [4,5]. In attempting to answer this question, it is first critical to define the time scale of interest because it is clear that the brain uses fundamentally different mechanisms to solve timing tasks across the range of microseconds used for sound localization to circadian rhythms [6–8]. Here, we focus on the time scale of tens of milliseconds to a few seconds. It is within this range that the most sophisticated and flexible forms of temporal processing reside, as it encompasses the temporal structure critical to the recognition and production of both speech and music. We review models of temporal processing and experimental data supporting the view that timing in this range relies on intrinsic neural properties, specifically that timing is among the most basic and general computations that neural circuits perform. This review focuses primarily on the electrophysiological experimental data and theoretical models; we do not carefully address behavioural and psychophysical experiments, which have been reviewed elsewhere [4,6,8–14].

## 2. Models of timing

Timing has been proposed to rely on a number of different potential mechanisms. These models cover a broad range of different neural substrates and vary dramatically in the level of biological detail and plausibility. Broadly speaking, these theoretical models of timing can be loosely categorized into four classes [8,15]: (i) spectral/delay line models; (ii) oscillator models; (iii) ramping models; and (iv) state-dependent network (SDN) models. We briefly describe the first three classes of models, and then describe the state-dependent models in more detail before reviewing the experimental studies of timing.

### (a) Spectral/delay line models

Some of the earliest models of temporal processing relied on the notion that representations of recent events were ‘buffered’ through delay lines. This view was strongly influenced by the delay line models of sound localization [16]. According to this model, interaural time differences on the order of tens of microseconds are computed in the auditory brainstem based on two mechanisms: delay lines and coincidence detectors. Delays are introduced as a result of axonal length, and when the delays are counterbalanced by the input time difference between them, neurons functioning as coincidence detectors report the relative timing of both inputs [16–18]. On longer time scales, conceptually similar models—sometimes referred to as tapped delay lines—operate under the assumption that different neurons are activated or receive inputs at different time delays [19–21]. For example, early cerebellar models emphasized the potential of parallel fibres (the axons of cerebellar granule cells) to function as delay lines on the order of tens of milliseconds—this notion has never received experimental support and it is generally acknowledged that the conduction time delays in the cerebellum are unlikely to contribute to timing on scales above tens of milliseconds. More generally, spectral models propose that there is a range (a spectrum) of different delays timing a range of different intervals [22].

Spectral models share the common principle that at least one variable or property of a neuron is set to a different value, which endows each unit with the ability to respond selectively to different intervals. In addition to axonal length [21], a wide range of different biological loci have been proposed to account for the delays, including kinetic constants of the metabotropic receptor pathway [23], the time constant of slow membrane conductances [24] and the decay time of inhibitory postsynaptic potentials (IPSPs) [25,26]. Spectral models have the advantage of encoding the time since the arrival of a stimulus by having different subsets of cells active at different times. Combined with simple learning rules, where a teaching or error signal modifies connections for only those cells that are active, it is possible for spectral models to learn outputs that are properly timed.

### (b) Oscillator models

When considering the mechanisms of timing, it is perhaps most intuitive to think in terms of devices that share the principles of man-made clocks. Indeed, the most influential model of timing is based on the notion of an oscillator that elicits pulses at certain intervals and a counter that integrates the pulses of the oscillator providing a linear metric of the passage of time [27,28]. Subsequent versions of clock-like models proved hugely valuable in guiding behavioural animal experiments [29–32], however the ‘standard’ internal clock model has not yet been expressed concretely in biologically realistic models.

More sophisticated oscillator-based models have proposed that timing arises from a population of elements oscillating at different frequencies [31,33,34]. These multiple-oscillator models have the advantage of not requiring integrating or counting the pulses in any of the oscillators, but rely on detecting specific ‘beats’ or synchronous patterns among the population of oscillators. This detection process can be performed by read-out neurons that detect the coincident activity of a subset of oscillators—corresponding to a specific point in time. While oscillations are a robust property of many different types of neurons, it remains to be determined whether stimuli can trigger a set of oscillators at different frequencies. Additionally, multiple-oscillator models have focused primarily on timing in the suprasecond range, rather than the subsecond range.

### (c) Ramping models

Ramping models propose that time is encoded in the approximately linear increase in the firing rate of neurons [35,36]. Experimental studies have conclusively established that some neurons can exhibit a more or less linear ramping of firing rate during timing tasks, and that the slope of the ramp can be modulated so that a particular rate is reached at a target time [37–42]. In this framework, a timed motor response can be generated when the firing rate of a neuron reaches a certain threshold value. The neural mechanisms underlying this ramping are not known, but have been proposed to be a product of either the intrinsic properties of neurons or the dynamics of local neural networks [35,36,43].

During timing tasks motor responses or expectation of an event are, of course, tightly coupled with elapsed timing. Thus, in many tasks there is a potential confound regarding whether ramping is actually encoding time or responsible for triggering a motor response (or representing the expectation of a reward). At least some data suggest that ramping neurons are coding expectation rather than absolute time [44]; furthermore, while upward ramping is seen during some types of timing tasks it is not observed in others [45].

### (d) State-dependent network model

SDN models propose that timing arises from the time-dependent changes in network state imposed by time-varying neural properties and the neural dynamics characteristic of recurrent circuits [8,15,46–48]. The SDN model is a prototypical example of an intrinsic model of timing, in that it does not rely on what most would consider specialized timing mechanisms. Similarly, this class of models is also local, that is, any cortical network could potentially process temporal information.

To understand how the state of a neural network might encode time, the dynamics of a liquid provides a useful analogy. A pebble thrown into a pond will create a spatio-temporal pattern of ripples, and the pattern produced by any subsequent pebbles will be a complex nonlinear function of the interaction of the stimulus (the second pebble) and the internal state of the liquid (the current pattern of ripples). Ripples thus establish a short-lasting and dynamic memory of the recent stimulus history of the liquid, and it is possible to estimate the amount of time elapsed based on the current state of the liquid.

In defining the state of a neural network, it is important to consider two general types of states: the active and hidden states [49]. (i) The *active state* at any given time point refers to the population of neurons that is currently firing. The active state thus represents the traditional spatio-temporal patterns of activity. (ii) The hidden states refer to the collection of time-varying neuronal and synaptic properties. These include slow synaptic currents, channel kinetics and short-term synaptic plasticity (STP). These properties are referred to as the ‘hidden’ state because they typically cannot be measured using conventional extracellular recording methods, but the effects of these time-dependent neural mechanisms are expressed at the level of firing properties when the network is ‘probed’ by a subsequent sensory stimulus. SDN models have typically focused on STP as the principal contributor to the hidden state of neural networks. STP refers to a form of use-dependent change in synaptic efficacy that is observed in almost all synapses [50,51]. As a result of STP, the second of two consecutive exciatitory postsynaptic potentials (EPSPs) can be larger (facilitation) or smaller (depression) than the first. In a sense, in the same manner that long-term potentiation provides a memory of coincident activity between groups of synapses that occurred minutes or hours in the past [52,53], STP provides a memory of an event that happened tens or hundreds of milliseconds ago.

The hypothesis that the dynamically changing active state of neural networks may encode time was first proposed by Michael Mauk in the context of the Marr–Albus–Mauk cerebellar model [54–56]. In this model, there is a dynamic turnover in the population of active granule cells as a result of the negative feedback loop between granule and Golgi neurons.

**Figure 1.**
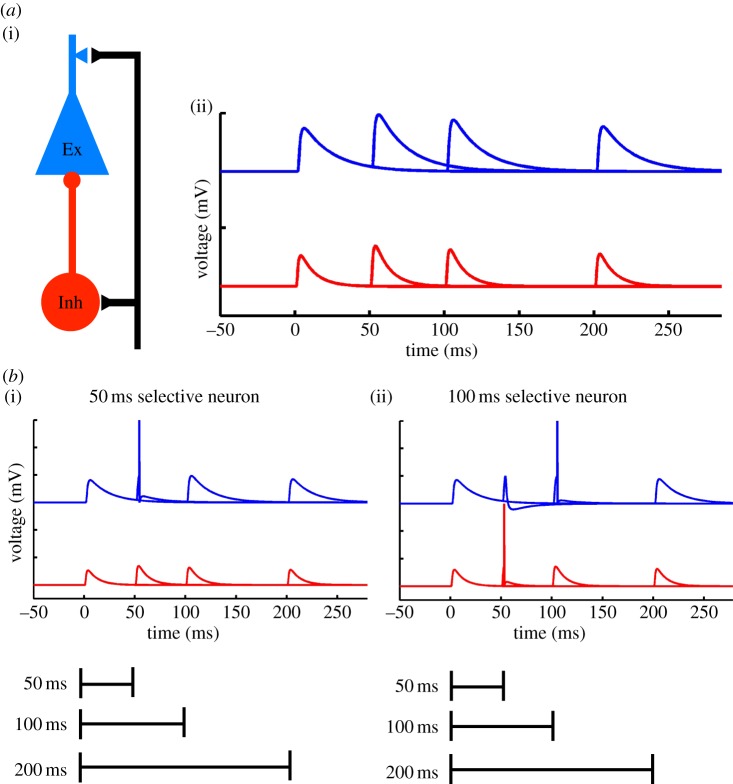
Interval selectivity based on short-term plasticity in simple circuits. (*a*) (i) Schematic of a feed-forward disynaptic circuit. Such circuits are almost universally observed throughout the brain. They are characterized by an input that excites both an inhibitory (Inh) and excitatory (Ex) neuron (for example, thalamocortical axons synapse on both excitatory and inhibitory neurons), and feed-forward inhibition (the excitatory unit receives inhibition from the inhibitory neuron). Each of the three synapses exhibits STP. (ii) Simulated paired-pulse facilitation (PPF) of EPSPs on to an Ex (blue) and Inh (red) neuron. (*b*) STP (the hidden state) can potentially be used to generate interval-selective neurons. Perhaps the simplest scenario is one in which both the excitatory and inhibitory neurons receive inputs that exhibit PPF. (i) An example of a 50 ms interval-selective neuron, where the Ex responds selectively to a 50 ms interval, simply because PPF peaks at 50 ms. (ii) An example of a 100 ms interval-selective neuron. Compared with (i), the strength of the excitatory weight onto both the Ex and Inh neuron has been increased. The spike in the Inh neuron at 50 ms ‘vetoes’ a spike in the Ex neuron, which responds to 100 ms because of the increase in the synaptic strength of the inputs (adapted from Buonomano [48]).

The concept that time may be encoded in the population of neurons was later generalized to cortical circuits and to incorporate the hidden state of neural networks. In a reduced circuit composed of a single excitatory and inhibitory neuron, it is easy to see how STP can potentially account for interval selectivity (figure 1). For example, if facilitation peaks at 50 ms, it is possible to adjust the synaptic weight so that a neuron will fire exclusively at 50 ms. Interestingly, in the context of simple disynaptic circuits composed of a single excitatory and inhibitory neuron, it is possible to tune the circuit so that the excitatory neuron fires selectively to a range of different intervals even though the temporal profile of STP remains the same [48]. As described below, experimental evidence supports the notion that interval selectivity in neurons arises from dynamic changes in the balance of excitation and inhibition imposed by STP.

**Figure 2.**
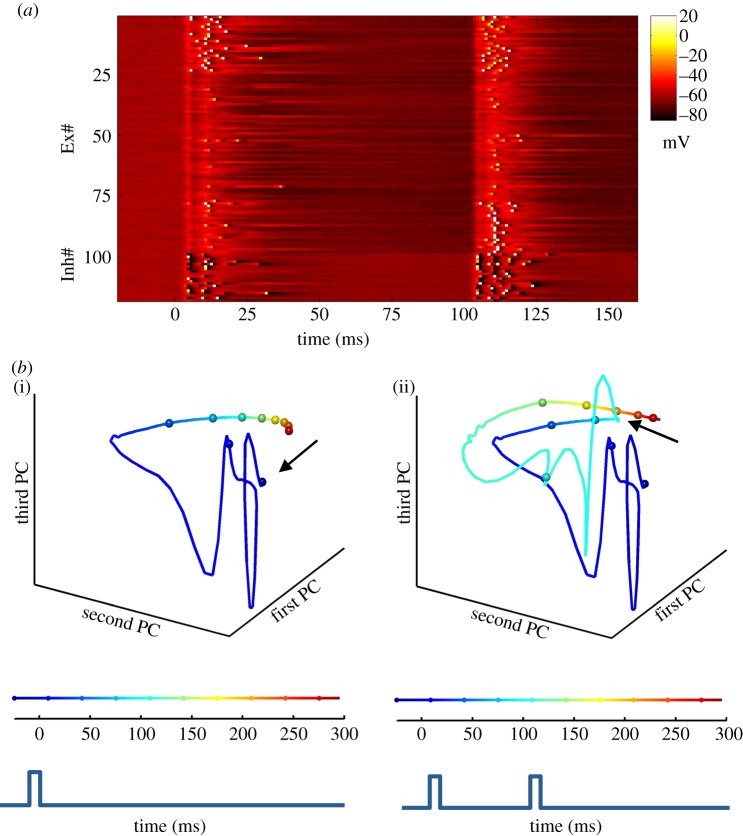
Example of interval-selective neurons in an SDN model of cortex. (*a*) Simulation of a recurrently connected network composed of 500 integrate-and-fire neurons. Each line represents the voltage of a single neuron in response to two identical events separated by 100 ms (representing two auditory tones). The first 100 lines represent 100 excitatory units (out of 400), and the remaining lines represent 25 inhibitory units (out of 100). Each input produces a depolarization across all neurons in the network, followed by inhibition. While most units exhibit subthreshold activity, some spike (white pixels) to both inputs or selectively to the 100 ms interval. The Ex units are sorted according to their probability of firing to the first (top) or second (bottom) pulse. This selectivity to the first or second event arises because of the difference in network state at *t* = 0 and *t* = 100 ms. (*b*) (i) Trajectory of the network in response to a single pulse. The state of the network can be represented by a trajectory that captures both the active and hidden states of the network. Principal component (PC) analysis is used to visualize the state of the network in three-dimensional space. There is an abrupt and rapidly evolving response beginning at the onset of the stimulus (*t* = 0), followed by a slower trajectory. The fast response is due to the depolarization of a large number of units (changes in the active state), while the slower change reflects the short-term synaptic dynamics (the hidden state). The speed of the trajectory in state-space can be visualized by the rate of change of the colour code and by the distance between the 25 ms marker spheres. Because synaptic properties cannot be rapidly ‘reset’, the network cannot return to its initial state (arrow) before the arrival of the second event. (ii) The trajectory in response to a 100 ms interval. Note that the first and second (arrow) presentation of the same stimulus produces different trajectory segments. In other words, the same input produced different responses depending on the state of the network at the arrival of the input (adapted from Karmarkar & Buonomano [57]).

We stress however, that the SDN model does not suggest that the timing that underlies complex temporal processing is solely the result of carefully tuned weights and STP in simple disynaptic circuits. Rather, the core proposal of SDN models is that temporal-selectivity is in part an inevitable consequence of the rich repertoire of time-dependent synaptic and neural properties embedded within large intricately connected recurrent neural networks. Indeed, as shown in figure 2, computer simulations demonstrate that in randomly connected simulated cortical networks, a percentage of the neurons exhibit interval selectivity [48,57]. Thus, the key notion is that complex neural circuits—in regimes in which excitation and inhibition are balanced—will naturally possess neurons that are selective to temporal features as an inevitable consequence of the dynamics of the network.

It is important to note that the different classes of models described above are not mutually exclusive. Timing and temporal processing encompass a very broad range of tasks requiring different computational needs. Thus, it seems likely that multiple mechanisms will contribute to timing. However, we focus here primarily on the SDN model, which we argue better accounts for the experimental evidence described below. We should also stress that the spectral, oscillator and ramping models are mostly limited to relatively simple forms of timing, for example interval discrimination. It seems unlikely that these models are sufficiently flexible and powerful to account for the timing necessary for speech, music or Morse code.

## 3. Temporal selectivity *in vivo*

The fact that many animals are capable of discriminating simple intervals or durations indicates that there are neurons in their brains that respond with at least some degree of selectivity to the temporal features of stimuli. There are numerous reports of temporally selective neurons. However, compared with the spatial features of sensory stimuli, such as position or orientation of visual stimuli, or frequency or intensity of auditory tones, very little is known about the location, development and mechanisms underlying temporally selective neural responses.

Some of the best examples of temporal selectivity have been performed in invertebrates and non-mammalian vertebrates. Extracting temporal information from acoustic stimuli is a crucial part of social communication of invertebrates and vertebrates. In some species, including frogs, electric fish and crickets, communication relies on relatively simple temporal codes in which information is conveyed by the interval between sensory events. Thus, these animals have provided an ideal model system to identify temporally selective neural responses and study the underlying neural mechanisms. Below, we discuss three examples of temporally selective neurons, in which the selectivity seems to arise from dynamic changes in the balance of excitation and inhibition imposed by STP.

**Figure 3.**
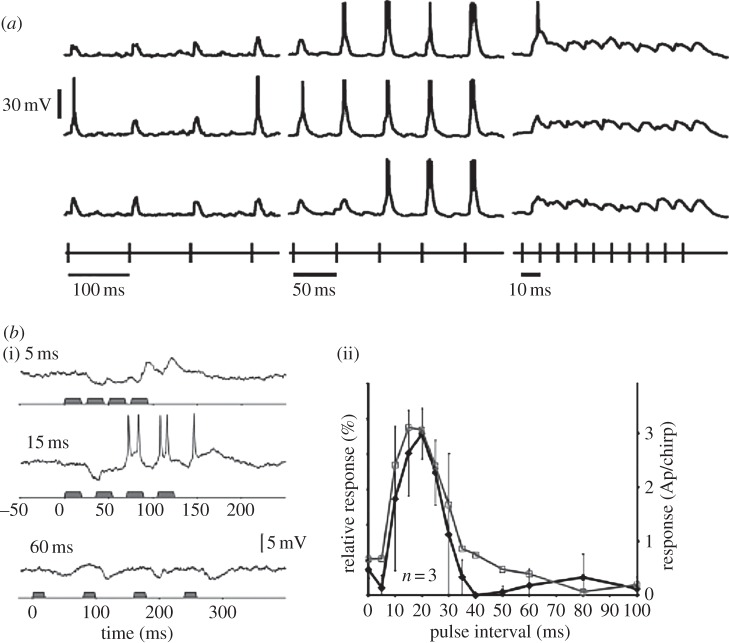
Example of temporally selective neurons in electric fish and crickets. (*a*) Voltage traces from a neuron in the midbrain of an electric fish. Each voltage trace represents the delivery of trains of electrical pulses presented at intervals of 100 (left), 50 (middle) and 10 ms (right). The rows represent three separate repetitions of each train. The electrical pulses were delivered in the chamber, picked up by the fish's electroreceptors and indirectly transmitted to the neuron in the exterolateral nucleus. This neuron was fairly selective to pulses delivered at intervals of 50 ms (adapted with permission from Carlson [59]). (*b*) (i) Spike activity from auditory brain neurons from female crickets were obtained in response to call songs of the male cricket. Voltage traces from an auditory neuron in response to songs with different pulse intervals. The three panels show traces obtained in response to songs where the inter-pulse interval was 5 ms (top), 15 ms (middle) and 60 ms (bottom). This particular neuron exhibited selectivity to temporal patterns with an inter-pulse interval of 15 ms. (ii) Temporal tuning of neural responses (black) and behavioural phonotactic (grey) responses obtained from three female crickets over a range of different pulse intervals (10–100 ms) (adapted with permission from Kostarakos & Hedwig [60]).

*Electric fish.* The temporal structure of electric pulses is an integral part of social communication in mormyrid electric fish [58]. The frequencies of pulses in the range of 10–100 Hz convey information about the temporal characteristics that encode species-specific communication. Intracellular recordings from electrosensory neurons in the brainstem of these fish have revealed selective responses to electrical pulses repeated at specific intervals, leading to high-frequency, low-frequency or bandpass neurons [59]. Figure 3*a* provides an example of a neuron that is tuned to respond to pulses presented at intervals of 50 ms, compared to intervals of 10 or 100 ms.

*Crickets.* Female crickets use the information contained in the temporal characteristics of the male cricket's song for phonotactic orientation [61,62]. Recordings from auditory neurons in response to different song patterns revealed the presence of a particular class of interneurons that respond selectively to pulses presented at specific intervals. For example, a subpopulation of neurons exhibited firing rate versus pulse interval tuning curves that closely mirrored the behavioural response which peaked at intervals of 15 ms. These neurons would not respond to a sequence of four pulses presented every 5 or 60 ms, but spiked when the four pulses were presented at 15 ms intervals (figure 3*b*) [60]. When the intervals between pulses were either too short (5 ms) or too long (60 ms), IPSPs were elicited to the first few pulses in the sequence followed by weak or negligible EPSPs to the subsequent pulses. When the pulse interval was optimal, the first few pulses were still dominated by pronounced IPSPs but the latter pulses in the temporal pattern elicited suprathreshold EPSPs accompanied by spikes.

*Frogs.* Some species of frogs also communicate through a temporal code in which the calls of different species are defined by their temporal structure [3,63,64]. Neurons in the auditory brainstem of these anurans have also been shown to respond selectively to the interval and number of pulses in a sequence. Short-term plasticity and the interplay of excitation and inhibition contribute to the interval selectivity observed in ‘interval-counting’ neurons [65]. The interval tuning of these auditory neurons is interesting in that they are tuned to respond only when a certain number of pulses with the optimal interval duration has been repeated. Hence, they are termed as interval-counting neurons because they show pronounced enhancement of excitation over a selective range of pulse repetition rates. Furthermore, if the temporal sequence of pulses contains a single non-optimal pulse interval (wherein the interval is too long or too short), then the interval-counting procedure is reset. A detailed study by Edwards *et al*. [65] examined the neural mechanisms of ‘interval counting’. They found that for a given neuron, when the number of pulses in a temporal sequence of optimal intervals exceeded a threshold, there was an enhancement of EPSPs often accompanied by spikes followed by subsequent inhibition.

Together, the above studies provide examples of neurons that respond selectively to the temporal structure of sensory stimuli. While the exact mechanisms underlying the interval selectivity are only partially understood, all of the above studies favoured interpretations based on dynamic changes in the balance of EPSPs and IPSPs imposed by STP—as proposed by the SDN model [48].

### (a) Temporally selective neurons in mammals

Neurons that respond selectively to the temporal and spatio-temporal structure of sensory stimuli have also been identified in the auditory system of birds [66–69] and mammals [70–76]. Identifying temporally selective neurons, also referred to as temporally combination sensitive neurons, is particularly challenging in mammals because of the complexity and multiplicity of auditory fields. A further problem relates to the combinatorial explosion of potential spatio-temporal stimuli to be explored—that is, even if a given neuron was highly selective to some specific spatio-temporal stimulus pattern, it is difficult to identify what that stimulus may be. Nevertheless, a number of studies have provided careful quantitative descriptions of neurons that respond preferentially when pairs or sequences of tones are presented in a given order with a specific interval between them [70,71,73–75,77]. These studies reveal a rich repertoire of cells that generally exhibit complex interactions between the frequencies and durations of the tones, and the interval between them. The mechanisms underlying these examples of interval and order selectivity have not been carefully examined but are broadly consistent with SDN models.

## 4. Timing and neural dynamics *in vitro*

The fact that interval- and order-selective neurons have been observed in a wide range of subcortical and cortical areas across many species favours the notion that timing may be a general and intrinsic property of neural networks. Perhaps the most rigorous prediction of models that propose that timing is an intrinsic computation is that timing may be able to be observed *in vitro*. That is, if neural networks are indeed intrinsically capable of timing, it may not only be possible to observe examples of timing *in vitro*, but also to, in a sense, ‘teach’ *in vitro* circuits simple temporal tasks. We now discuss studies that have attempted to ask and answer this question experimentally.

These studies focus on examples of timing that may rely on the active and hidden states of neural networks. When timing relies on the active states of neural networks, the notion is that autonomous dynamics changes the population of active neurons. A number of *in vivo* experimental studies support the notion that time-dependent changes in the active state of neural networks may encode time [78–82]. We focus, however, primarily on *in vitro* studies on neural dynamics and timing because they provide a tractable approach to understand the underlying mechanisms. And because, as mentioned, *in vitro* timing would provide strong support to the notion that timing is a basic and general computation of neural networks.

### (a) Neural dynamics *in vitro*

Several *in vivo* and *in vitro* studies have shown that the richly interconnected circuits in the cortex exhibit complex dynamic patterns of activity [83–90]. This dynamics can be studied through intracellular recordings from single neurons, extracellular recordings from multiple neurons or multi-cell Ca^2+^-imaging; and methods have revealed that complex yet reproducible patterns of activity appear to be a general and widespread regime of recurrent neural circuits.

**Figure 4.**
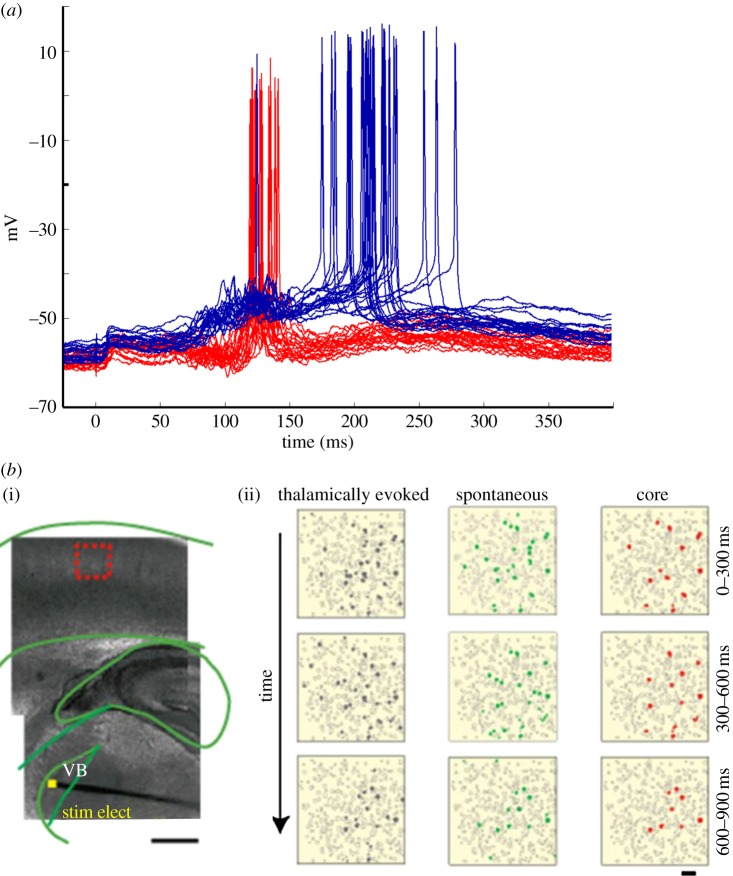
Neural dynamics *in vitro* cortical networks. (*a*) Simultaneous intracellular recordings from two neurons in organotypic cortical slices in response to a single electrical stimulus at *t* = 0 ms. The polysynaptic activity in both neurons provides a measure of the recurrent neural network activity. The fact that both neurons fire with a high probability within specific time windows across trials (overlaid traces) demonstrates the reproducibility of the network dynamics and the presence of temporal information. Data shown consist of 20 superimposed traces obtained from the red and blue neuron (adapted from Buonomano [48]). (*b*) (i) Simultaneous recordings from several L-IV neurons using calcium imaging shows stimulation-induced spatio-temporal patterns of network activity generated as a result of internal dynamics of the network. Light microscope image of a somatosensory thalamocortical slice preparation. Stimulating electrode (stim elect) is placed in the ventral basalis (VB) as indicated by the yellow square and recordings were made from the region indicated by the red square. (ii) Representative movies obtained from thalamically induced network activation and spontaneous network activity. The rightmost column of panels shows core sample movies that represent neurons that are coactive during both electrical stimulation and spontaneous network activity (reproduced with permission from Luczak & MacLean [85]).

*In vitro* whole-cell recordings in both acute and organotypic slices demonstrate that in addition to a short-lasting monosynaptic EPSP, external stimulation can elicit long-lasting polysynaptic responses. This polysynaptic activity reflects the internal dynamics of local recurrent networks. Figure 4*a* illustrates an example of evoked polysynaptic activity in simultaneously recorded neurons in an organotypic cortical slice. Note that even the intracellular polysynaptic activity recorded in a single neuron provides a measure of overall network activity in that the subthreshold PSPs reflect a read-out of the subpopulation of neurons connected to the recorded cell. In this example, is it also clear that across trials the two neurons tend to fire at different time points (129 and 205 ms after the stimulus). In a manner of speaking, these neurons and the networks they are embedded in can be said to encode time. Specifically, if a neuron reliably fires during some time window after the stimulus, this neuron contains information about how much time has elapsed since the stimulus.

Simultaneous recordings from ensembles of L-IV neurons using Ca^2+^-imaging also reveal that electrical stimulation can elicit spatio-temporal patterns of network activity. For example, MacLean *et al*. [84] have shown that acute cortical slices can exhibit reproducible patterns of activity (up states) in response to thalamic stimulation that persists for hundreds of milliseconds or even seconds beyond the duration of the stimulus. Interestingly, this particular study also found that subsets of L-IV cells that participated in thalamically evoked network responses also participated in spontaneous network activity (figure 4*b*). Together, these results suggest that cortical networks *in vitro* can generate reproducible patterns of activity that can be thought of as a neural trajectory—e.g. in neural state-space, the activity of each neuron in a network corresponds to an axis in high-dimensional space and one can plot the changing activity of the network as a trajectory. As is the case with the trajectory of any dynamical system that is moving through state-space, it naturally encodes time, functioning in effect as what has been called a population clock [5,91]. Critical to the potential ability of these patterns to be able to encode time is that they must be reproducible across different trials. The degree of variability across trials places strong constraints on the ability and accuracy of such patterns to potentially encode time.

### (b) State-dependent responses *in vitro*

A number of *in vitro* studies have attempted to ask whether *in vitro* networks can encode information about time or previous stimuli in a state-dependent fashion [92–94]. One group has examined this question in rodent hippocampal slices wherein different stimuli were presented to hippocampal slices via stimulation of electrodes in the perforant pathway [93,95]. Responses to stimuli were recorded during intracellular recordings of up to three simultaneously recorded hilar neurons. It was demonstrated that the different patterns of activity in three hilar neurons could be used to decode which of four perforant path electrodes had been stimulated. This was true even 15 s after the stimuli had been presented—indicating that ongoing activity patterns within the network provided a memory of the stimulus. More importantly, however, when sequences of four stimuli (A, B, C and D) were presented, e.g. ABCD × DCBA, there was also a robust context- or state-dependent code that allowed, for example, one to determine whether A had been presented by itself or preceded by other stimuli.

A related *in vitro* study, performed in dissociated cultures on multi-electrode arrays, also examined whether a population of neurons could encode the sequences of different input patterns to the network [94]. In this study, neurons were transfected with channelrhodopsin (ChR) and different ‘visual’ patterns generated using an optical grid that stimulated different subsets of ChR-positive neurons, serving as different ‘sensory’ inputs. The response of the network was quantified based on the extracellular recordings of dozens of units. The results demonstrated that in response to the presentation of stimulus pairs, such as AC or BC (each separated by intervals of up to 1 s), it was possible to look at a population response elicited by stimulus C and determine whether it had been preceded by A or B. In other words, the network activity in response to C implicitly encoded the context in which it was presented. Because network activity often faded back to baseline within a 1 s time window, it is suggested that the ‘memory’ of the previous stimulus is represented in the hidden state of the network.

While relatively few studies have examined the capacity, complexity and temporal features of stimuli that *in vitro* networks can encode, there is now significant evidence that even *in vitro* networks are capable of encoding order and temporal information as predicted by SDN models. Specifically, on the subsecond time scale, the population response to a stimulus can not only encode the identity of the current stimulus but that of the previous stimulus as well.

## 5. Neural dynamics and timing plasticity *in vitro*

The studies above reveal that recurrent networks can produce complex dynamics in response to a brief stimulus and encode information about stimulus order and time. However, it is not clear whether these networks were in some sense ‘designed’ to process temporal information, or in contrast, the observed timing was essentially a product of random activity patterns. A particularly valuable approach to determine whether recurrent neural circuits are in effect ‘designed’ for temporal processing would be to establish whether they can ‘learn’ to process temporal information. Here, we use the term ‘learn’ in a general sense and ask whether the neural dynamics of circuits exposed to a specific temporal pattern adapt in a manner that would improve the processing of the experienced pattern.

**Figure 5.**
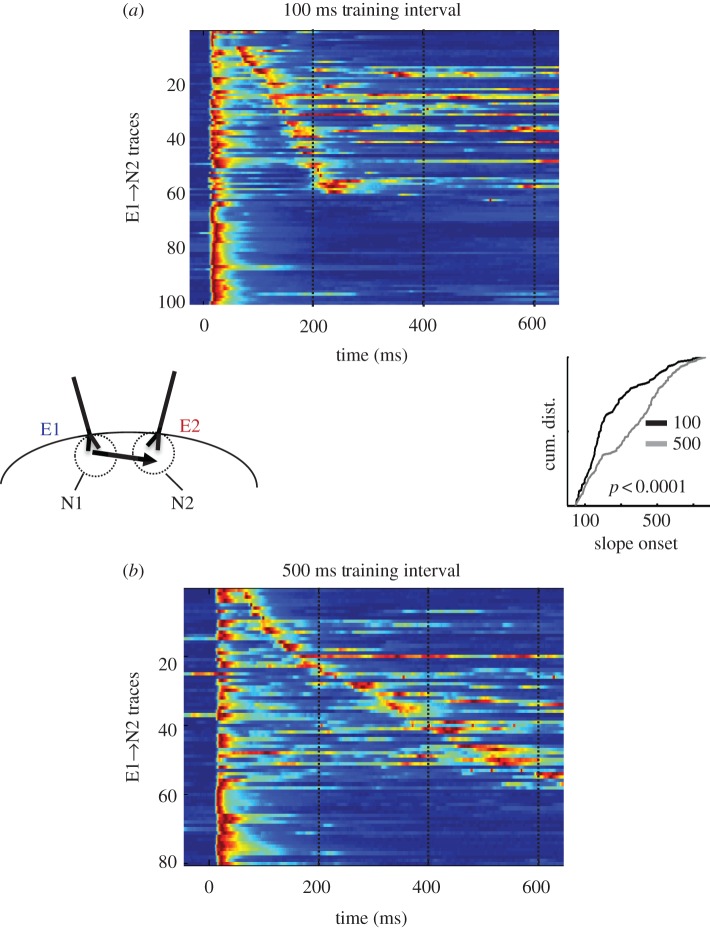
An example of an *in vitro* analogue of interval learning. Organotypic cortical slices were stimulated for 2 h (while in the incubator) with either a 100 ms training interval (*a*) or 500 ms interval (*b*). After training, whole-cell recordings were performed in neurons near the E2 electrode (left inset). Test stimuli consisted of single pulses to electrode E1. The coloured voltagegrams in (*a*,*b*) show all the data (all traces across all cells) sorted according to the timing of the polysynaptic response (if present). The voltage traces are normalized to their peak. The cumulative distribution of the polysynaptic events shown was significantly earlier in the 100 ms (black) compared with the 500 ms (grey) group (right inset) (adapted from Johnson *et al*. [97]).

One approach towards answering this question has been to ask whether the dynamics in *in vitro* cortical cultures can be sculpted by experience [96]. Cortical cultures were ‘implanted’ with a pair of electrodes that were used to provide structured ‘sensory’ input to the network. In the initial experiments, both electrodes were synchronously stimulated for 2 h (every 10 s), whereas in the second group, both electrodes were stimulated with a 100 ms interval: electrode E1 was activated at *t* = 0 and electrode E2 at *t* = 100 ms. Thus, the pathways in both groups received the same amount of total stimulation but differed in the temporal pattern they were exposed to. After training, whole-cell recordings were made from supragranular pyramidal neurons near E2 and the responses to E1 were analysed. The results demonstrate that there was significantly more polysynaptic activity in the 100 ms group than in the synchronous group. Furthermore, there was some clustering of polysynaptic responses evoked by E1 at around 100 ms. One interpretation of these data is that the cells near E2 ‘expected’ or anticipated a stimulus 100 ms after E1, and the network was performing a form of temporal pattern completion. This was further tested by training slices with either a 100 or 500 ms interval. As shown in figure 5, a comparison of evoked polysynaptic activity revealed a significant difference in the timing of the distribution of polysynaptic events. Specifically, the temporal profile of this polysynaptic activity reflected the interval used during training [97]: more short- and long-latency events in the 100 and 500 ms groups, respectively. Additionally, ongoing experiments have further explored the ability of *in vitro* networks to adapt to experienced intervals by combining electrical and optogenetic stimulation. In these experiments, an electrical pulse was followed by an optical stimulation of a subset of neurons transfected with the light-activated cation channel ChR. In one group there was a 100 ms interval between the electrical stimulus and light, and in the other a 500 ms interval. After 2 h of training, whole-cell recordings from the ChR-positive neurons revealed that there was a significant difference in the timing of the distribution of polysynaptic events: there was a larger proportion of late events in the 500 ms group compared with the 100 ms group [98].

Experience-dependent changes in network dynamics have also been observed *in vivo* preparations. Aizenman and co-workers [99] demonstrated that in *Xenopus* tadpoles, stimulation of the optic nerve elicited firing patterns that lasted hundreds of milliseconds in neurons in the optic tectum. These long-lasting responses were in part generated by the recurrent circuitry within the optic tectum, and interestingly the duration of the activity was influenced by experience. Presenting visual flashes at intervals of 150 or 400 ms for 4 h before recording sessions revealed different distributions of the spike times: median spike latencies of 73 and 106 ms, respectively.

A related *in vivo* study in the visual cortex of rats revealed experience-dependent changes of light-evoked neural activity in the context of an associative learning task [100]. During a training phase, rats received rewards at different delays (more specifically after a specific number of licks required for the reward) in response to flashes to the left or right eye. Extracellular recordings in V1 revealed that a significant percentage of neurons exhibited responses that peaked over a second after the stimulus offset and at approximately the expected time of the reward (even in the absence of the reward). These responses were specific to one eye and matched the delay associated with that eye, thus providing strong evidence that the neural dynamics was learned. In a subsequent study, the authors examined the contribution of neuromodulators to this form of associative learning task and demonstrated that changes in the temporal dynamics can also be observed in acute visual cortex slices [101]. Slices were exposed to a conditioning protocol that consisted of pairing brief electrical stimulation followed by a puff of a cholinergic agonist 0.5, 1 or 1.5 s after the electrical stimulus. After this training protocol, extracellular or intracellular recordings were performed in layer 5 neurons. The results revealed that the duration of the neural activity evoked by a single electrical stimulus was proportional to the interval used during training. That is, spiking activity was short lasting in the slices trained with a 0.5 s interval, and long lasting in slices trained with a 1.5 s interval. This study demonstrated that acute slices can ‘learn’ to fire with different temporal profiles and suggests that cholinergic input can provide a reinforcing signal that is required for this form of temporal plasticity.

The above examples demonstrate that network dynamics undergoes experience-dependent plasticity. Specifically, the duration or dynamics of neural responses within neural circuits can be shaped by the experience in a manner that should facilitate the processing of the temporal patterns the network experienced. Together, these findings support not only the hypothesis that recurrent neural circuits, and cortical circuits in particular, are capable of encoding time, but also that there are mechanisms in place that allow such circuits to ‘learn’ the temporal structure of the stimuli they are exposed to.

## 6. Conclusion

The temporal components of different sensory, motor, cognitive and learning tasks often require different levels of accuracy, precision, flexibility (e.g. the time it takes to ‘reset’ a clock between tasks) and complexity (e.g. simple intervals or complex patterns). Thus, it would seem that the sheer diversity of sensory and motor tasks that require timing hints that there may be multiple mechanisms operating in parallel. For example, a simple interval-timing task may require animals to time the interval between a cue and a response, expected reward or unconditioned stimulus, whereas interpreting Morse code or playing the piano requires the decoding and generation of very complex temporal patterns. While simple interval timing could rely on many different potential mechanisms including ramping firing rates or neural oscillators, it seems unlikely that complex temporal tasks rely on such mechanisms. Here, we have focused on a general framework that could potentially contribute to both simple and complex forms of temporal processing. Within this SDN framework, temporal computations arise from time-dependent changes in cellular or synaptic properties (the hidden state) and dynamic changes in the population of active neurons within recurrent neural circuits (the active state).

To date, the neural basis of temporal processing has proved to be a challenging problem. This is in part a product of the relative difficulty in identifying the brain regions underlying temporal processing, and reliably recording from temporally selective neurons. Consider, the relative ease with which neuroscientists can identify neurons that respond to the touch of a specific whisker in a rodent or a visual neuron that responds to a specific orientation. In contradistinction, even though numerous reports have identified instances of cortical neurons that respond selectively to temporal features [70–75], it is not possible to predict in advance which neurons are temporally selective or anticipate their preferred temporal features. This somewhat stochastic nature of temporal-selectivity is, however, what SDN models predict: that some percentage of neurons will fire selectively to order, interval and duration, but which ones do is in part a stochastic product of the connectivity and synaptic properties of the local circuitry. Indeed, computer simulations of randomly connected recurrent cortical networks that exhibit STP reveal that some percentage of the units exhibit temporally selective responses, but as a result of the complexity and randomness in the network it is hard to ascertain *a priori* which units will exhibit temporal-selectivity [46,48,57,102].

Whether or not STP and intrinsic dynamics of recurrent circuits account for timing in the subsecond range remains an open question. However, the *in vivo* studies described above in different areas of the invertebrate, frog, fish, bird and mammalian nervous system strongly favour the hypothesis that timing on the scale of tens of milliseconds to a few seconds is an intrinsic property of neural circuits, as opposed to specialized computations performed by some dedicated brain area [4]. Furthermore, we suggest that observations of timed responses in *in vitro* circuits, together with evidence that *in vitro* circuits adapt to the temporal structure of experienced temporal features, offer some of the best evidence for the intrinsic timing hypothesis. Thus, although the mechanisms underlying the diverse forms of temporal processing the brain performs remain to be elucidated, we believe that there is sufficient cumulative evidence to conclude that at least some forms of timing on the subsecond scale are a product of intrinsic mechanisms—that is, they are a general and inherent computation of neural circuits, as opposed to a product of dedicated or centralized neural circuits.
